# Partial masculinization of *Aedes aegypti* females by conditional expression of Nix

**DOI:** 10.1371/journal.pntd.0010598

**Published:** 2022-07-01

**Authors:** Bianca B. Kojin, Emma Jakes, James K. Biedler, Zhijian Tu, Zach N. Adelman

**Affiliations:** 1 Department of Entomology and Agrilife Research, Texas A&M University, College Station, Texas, United States of America; 2 Department of Biochemistry and Fralin Life Science Institute, Virginia Tech, Blacksburg, Virginia, United States of America; International Centre for Genetic Engineering and Biotechnology, INDIA

## Abstract

**Background:**

*Aedes aegypti*, the main vector of dengue, yellow fever, and other arboviruses thrives in tropical and subtropical areas around the globe putting half of the world’s population at risk. Despite aggressive efforts to control the transmission of those viruses, an unacceptable number of cases occur every year, emphasizing the need to develop new control strategies. Proposals for vector control focused on population suppression could offer a feasible alternative method to reduce disease transmission. The induction of extreme male-biased sex ratios has been hypothesized to be able to suppress or collapse a population, with previous experiments showing that stable expression of the male determining factor *Nix* in *A*. *aegypti* is sufficient to convert females into fertile males.

**Methodology/Principal findings:**

Here, we report on the conditional expression of *Nix* in transgenic *A*. *aegypti* under the control of the tetracycline-dependent (Tet-off) system, with the goal of establishing repressible sex distortion. A masculinization phenotype was observed in three of the seven transgenic lines with females exhibiting male-like long maxillary palps and most importantly, the masculinized females were unable to blood feed. Doxycycline treatment of the transgenic lines only partially restored the normal phenotype from the masculinized transgenic lines, while RT-qPCR analysis of early embryos or adults showed no correlation between the level of masculinization and ectopic Nix expression.

**Conclusions/Significance:**

While the conditional expression of Nix produced intersex phenotypes, the level of expression was insufficient to program full conversion. Modifications that increase both the level of activation (no tet) and the level of repression (with tet) will be necessary, as such this study represents one step forward in the development of genetic strategies to control vector-borne diseases via sex ratio distortion.

## Author summary

Morbidity and mortality from vector-borne diseases are an increasing public health problem. Approaches to reduce mosquito abundance to control pathogen transmission, known as population suppression, have been under development in an attempt to decrease the burden imposed by such diseases. One proposed strategy to collapse vector populations relies on skewing the sex ratio equilibrium to compromise fertility. Based on that, this research demonstrates the conditional expression of the male determining factor of the mosquito *Ae*. *aegypti* with the goal of establishing repressible sex distortion. Although full conversion of females into males was not achieved, a masculinization phenotype was observed in some transgenic lines, providing the foundation for the development of a new category of genetic vector control strategy via sex ratio distortion.

## Introduction

The sex ratio in most sexually reproducing organisms is in equilibrium when male and females are conceived, meaning that an individual spends the same amount of energy generating an equal proportion of both sexes [1]. When the ratio is different than the equilibrium the overall population’s fertility can be compromised. This is particularly interesting as a genetic control strategy aiming at population suppression of disease-vector species. Hamilton [2] suggested that mosquito populations could be eradicated using genetic sex ratio distorters, with the idea that a decline in female numbers would cause a decline in the population size that would eventually lead to a collapse.

Sex determination is the developmental programme that determines the male or the female pathway [3]. The genetic mechanisms underlying sex determination in insects are not conserved, especially the upstream instructive signals (reviewed in [3,4]). The three known male determining factors that govern the sex-determination cascade in mosquitoes are *Nix* in *Aedes aegypti* [5], *gYG2/Yob* in *Anopheles gambiae* [6–8], and *Guy1* in *Anopheles stephensi* [9]. These three genes are not related, making the development of systems for sex conversion in vectors a tailored process.

It has been shown that the ectopic expression of Nix under the strong Polyubiquitin promoter resulted in masculinized females [5]. Moreover, transgenic *Aedes aegypti* stably expressing *Nix* under its own promoter was sufficient to fully convert females into males, although they were unable to fly due to the absence of a myosin heavy-chain gene located in the M-locus [10]. These results serve as a foundation for the generation of conditional male-only strains that can be used in control strategies. In this case, an off-conditional system is required that allows bisexual maintenance of the strain in the rearing facility. When preparing for field release, male-only progeny selection can be performed on-demand, by removal of a blocking agent that suppresses expression of the *Nix* transgene.

The Tet-off system has proven to be an effective system to conditionally express proteins in different organisms [11–13]. In mosquitoes the Tet-system was used to modulate protein expression in pericardial cells and hemocytes of *Anopheles stephensi* [14], and in *Aedes aegypti*, it was used in the late-acting lethal genetic system of the transgenic line OX513A [15], and in the repressible female-specific flightless phenotype [16], both designed for use in a system called RIDL (Release of Insects carrying a Dominant Lethal gene) that focus on mosquito control.

Here we present the generation of *Aedes aegypti* transgenic lines that conditionally express *Nix* using the Tet-off system. Three of seven transgenic lines displayed a masculinization phenotype exhibiting long maxillary palps, diminutive body size and most importantly they were unable to blood feed. Transcript analyses reveled that those differences in the levels of masculinization did not correlate with levels of transgene expression among transgenic lines and the incomplete conversion is likely linked to low levels of transgenic *Nix* transcript accumulation in comparison to the endogenous in wild type mosquitoes. Though a fully conditional Nix-expressing strain remains to be established, this work demonstrates a significant step towards a male-only production system, for the purpose of generating field-release males for population control or other applications requiring male selection.

## Methods

### Assembly of Nix donor plasmid

**Fig 1:**
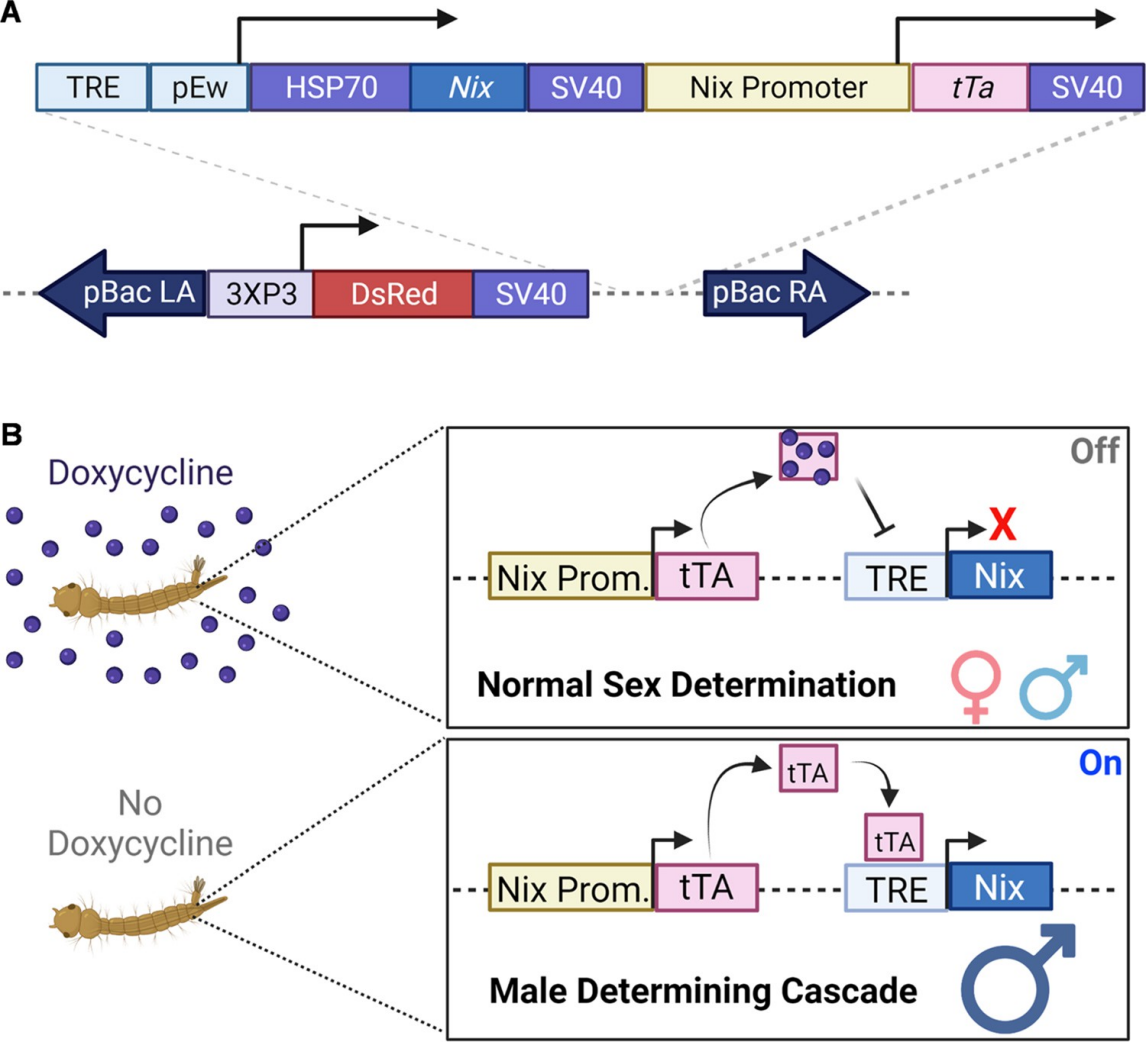
(**A**) A schematic of the pTRE-Nix/NixPro-tTA transformation construct and its landing site on the genome of transgenic lines. PiggyBac right arm (pBac R. A.) and left arms (pBac L. A.) are indicated, along with the 3XP3-DsRED marker cassette. The dotted lines represent sequence from the plasmid backbone. The pTRE-Nix/NixPro-tTA construct introduced another two genes: 1) The *P* element week promoter (pEw) driving the expression of the Nix protein. Upstream the promoter is the tetracycline responsive element (TRE) and downstream is the leader sequence of the heat shock protein 70 (HSP70). 2) The Nix promoter sequence driving the expression of the tetracycline Trans activator (tTA). All three genes on this construct have the 3’UTR from the simian virus 40 (SV40). (**B**) Schematic representation of the Tet-off system. Nix promoter (Nix Prom.). Illustrations were created using Biorender.com through a license to Texas A&M University.

The piggyBac donor plasmid [17] with the 3xP3-DsRed fluorescent protein marker was used as a backbone for the transgenic cassette (Fig 1A). The transgenic cassette consisting of the tetracycline transactivator (tTA) [18] driven by the *Nix* promoter/upstream sequence [10], and the tetracycline response element (TRE) [18] driving expression of the *Nix* protein [5], were synthesized and cloned into the backbone (Epoch Life Science, Missouri City TX, USA). See GeneBank accession number OM372429 for plasmid sequence.

### Mosquito rearing and transformation

*Ae*. *aegypti* Liverpool strain (LVP) were maintained at 28°C, 60–70% humidity and 14h light/ 10h dark cycle in chambers in an insectary. Blood feeding was performed exclusively on defibrinated sheep blood (Colorado Serum Company, Denver CO, USA) using an artificial feeding system for maintenance. The generation of *Ae*. *aegypti* transgenic lines was performed as described previously [19]. Briefly, preblastoderm embryos were microinjected with a mixture of pTRE-Nix/NixPro-tTA donor plasmid at 0.5μg/μL and the helper plasmid (pHSP-Bac) [20] at 0.3μg/μL using FemtoJet equipment (Eppendorf, Hamburg,Germany) and pulled borosilicate glass capillaries (World Precision Instruments, Sarasota, USA). Females from surviving injected embryos were pooled into ~20 individuals per cage and backcrossed to LVP in a 1:1 ratio, while males were mated with LVP females in a 1:5–10 ratio. The G_1_ progeny from injected embryos were screened for DsRed during larval stages. Transgenic lines obtained from female and male pools were identified by the letter P and F respectively, followed by a number representing the cage they came from (e. g. P5 or F3). All experiments were performed with transgenic heterozygous mosquitoes.

### Splinkerette PCR

Splinkerette PCR was performed as described in [21]. Briefly, Genomic DNA was extracted from the transgenic mosquitoes using the NucleoSpin Tissue kit (Macherey-Nagel, Duren, Germany) and digested overnight with BstYI. The annealed splinkerette oligonucleotides were ligated into the digested DNA in the presence of T4 ligase (New England Biolabs, Ipswich, USA) and used as a template for a nested PCR following the cycling parameters: 98°C for 75 sec; 2 cycles of 98°C for 20, 64°C for 15 sec; 30 cycles of 98°C for 20 sec, 64°C for 15s, 72°C for 2 min, and 1 cycle of 72°C for 7 min. The second round of PCR used 0.5 μL from the first PCR as a template and cycling parameters were as followed: 98°C for 75 sec; 30 cycles of 98°C for 20 sec, 66°C for 15s, 72°C for 90 sec, and 1 cycle of 72°C for 7 min. Amplification products were gel extracted and sequenced. Vector Base (http://www.vectorbase.org [22]), was searched for sequences corresponding to the junctions between transposon landing sites on *Ae*. *aegypti* genome and transposon arms using the blastn tool.

### Imaging, maxillary palps and body measurements

Mosquitoes were CO_2_ anesthetized and placed on top of a petri dish containing a filter paper and placed on ice. Bright field images were obtained using AmScope (version x64, 3.7.58492015) software. Maxillary palps were measured from the division of the head to the tip of the palp, and abdomen length was used as a proxy for body size and was measured from the tip of the last segment in the abdomen until the division of the abdomen to the thorax in the first segment, both using the line selection tool of ImageJ software (version 1.53g).

### Blood feeding success

Pre-mated females 5–7 days old were starved for 24hrs and then offered a blood meal in an artificial feeding system or in a cotton ball soaked in defibrinated sheep blood (Colorado Serum Company, Denver CO, USA) for 30 min. For feeding with soaked cotton, the blood was warmed in a water bath to 37°C prior to being added to the cotton ball, with the feeding occurring in a temperature controlled chamber (28°C) to delay the cooling of the blood. After the blood meal, females were CO_2_ anesthetized and scored for blood presence in the abdomen. Females with any amount of blood were considered blood fed.

### Sex ratio

To assay the sex ratio from the transgenic lines, transgenic males were backcrossed to LVP females and the progeny (transgenic and wild type) were reared together until screening for the transgenesis marker and then reared separately through adulthood when adult females and males total number were counted.

### Survival assay

About a hundred females and males from each transgenic line and LVP were observed for 38 days and dead mosquitoes were removed from the cages and recorded daily.

### Reverse transcription quantitative PCR (RT-qPCR)

RNA was extracted from female and male mosquitoes from both transgenic and wild-type strain from either the entire adult body, dissected tissues, or embryos. In tissue-specific expression experiments, 25–30 maxillary palps and antennae were dissected per replicate from LVP males and transgenic females. For embryo collection, females from transgenic and LVP controls were blood fed and after 72h they were placed in tubes with access to wet cotton wool and a disk of filtered paper on top and allowed to lay eggs for 20 min. After that, females were released into cages and at least 50 embryos were collected at different time points and used directly for RNA extraction. Only 5–7 days old adult mosquitoes were used in all experiments. Total RNA was extracted using TRIzol (Thermo Fisher Scientific, Waltham, USA) following the manufacturer’s protocol. The isolated RNA was treated with ezDNAse (Thermo Fisher Scientific, Waltham, USA) and quantified on a SpectraMax spectrophotometer (Molecular Devices, Sunnyvale, CA). One microgram of treated RNA was used to synthesize cDNA using SuperScript IV VILO Master Mix (Thermo Fisher Scientific, Waltham, USA) that contains both oligo (dT)18 and random hexamer primers also following the manufacturer’s protocol.

Reverse transcription quantitative PCR (RT-qPCR) was carried out with SsoAdvance Universal SYBR Green Supermix (BioRad, Hercules, USA) on a CFX96 Touch Real-Time PCR Detection System (BioRad, Hercules, USA). The primers used were designed on Primer 3 server (v.0.4.0) [23,24] with the amplification products not longer than 135 bp. Amplification efficiency was verified to be 0.9–1.0 in a reaction using DNA plasmid as a template. Reactions were performed with 1:50 diluted cDNA in technical triplicates for whole body or 1:20 for dissected tissues, with the primers listed in S1 Table, following the cycling parameters: 30 s at 95°C, 45 cycles of 15 s at 95°C, 15 s at 60°C and 10 s at 72°C, and melt curve analyses at 65–95°C. The dCT method was used to calculate expression relative to the rpS7 gene [25].

### Phenotype recovery assay and doxycycline treatment

Transgenic females mated with LVP males were offered sucrose containing 50μg/ml of doxycycline (Dox) *ad libitum* until blood feeding. After 72 hrs a small beaker containing water with 50μg/ml of Dox was placed in the cage for egg collection. Eggs were hatched and placed in containers containing 1L of distilled water, 50μg/ml of doxycycline (Dox) and 2ml of *E*. *coli* cells (SURE cells, Agilent, Santa Clara, USA) O.D. 0.6–1. SURE *E*.*coli* cells resistant to tetracycline and analogs were used to help alleviate the detrimental effects of antibiotic treatment on larval development, since previous work has shown that even a single bacterial species, including *E*.*coli*, is sufficient to rescue nutritional defects that larvae experience when completely axenic. [26]. Water from larval pans was changed every other day with the addition of new Dox and SURE cells until adult emergence. Controls consisted of mosquitoes reared in the absence of Dox but in the presence of SURE and non-transgenic controls were reared together with transgenic larvae until they were screened for the presence of the fluorescent marker. After that they were reared in different containers but following the same regimen as transgenic mosquitoes. Adult mosquitoes were used for maxillary palp and body measurements, blood feeding success experiments, mortality curves and sex ratio of transgenic mosquito lines.

### Statistical analysis

Binomial test was used to access statistical significance of the sex ratio of the transgenic lines and Tukey’s test was used in body size and maxillary palp length of transgenic lines in the absence of doxycycline. The fecundity, fertility treated or not with doxycycline, and maxillary palp length in doxycycline treated mosquitoes was evaluated by Mann-Whitney U test. Log-rank test was used to determine P values and statistical significance was determined after the Bonferroni method to correct for multiple testing in survival curves. In RT-qPCR experiments, transgenic and endogenous *Nix* expression profile in transgenic mosquitoes were tested by one-way ANOVA, and multiple t test was used to verify the impact of doxycycline on transgenic *Nix* expression. All analyses were performed using GraphPad Prism Version 7.02 and P values < 0.05 were considered statistically significant.

## Results

### Generation of Aedes aegypti conditionally expressing Nix through the Tet off system

We sought to evaluate the conditional expression of the dominant male-determining factor Nix using the Tet-off system in transgenic *Ae*. *aegypti* mosquitoes. The donor plasmid (Fig 1) contained three expression cassettes between the piggyBac inverted repeats: the tetracycline responsive element (TRE) preceding the P element weak promoter driving the expression of *Ae*. *aegypti* Nix, the *Nix* promoter driving the expression of the tTA, and the marker gene 3XP3-DsRed. Our expectation was that in transgenic mosquitoes reared in the absence of tetracycline or its analogs, tTA (driven by the *Nix* promoter) would be expressed before sex is established, which in turn would bind the TRE, activating *Nix* expression. Therefore, mosquitoes carrying this transgene should initiate the male determining cascade even in females that lack the endogenous *Nix* gene, converting genetic females to phenotypic males. In individuals reared in the presence of tetracycline or its analogs, the transgene would be turned off as tTA binds with higher affinity to the antibiotic resulting in normal individuals, and a regular sex ratio 1:1 males to females (Fig 1B).

The donor plasmid along with a helper plasmid that encodes the piggyBac transposase was injected into 2,316 preblastoderm *Ae*. *aegypti* embryos. From these injected embryos, 509 adults were backcrossed to LVP and G_1_ progeny screened for the presence of the marker gene. A transformation rate of 1.4% was reached resulting in the generation of 7 transgenic lines (Table 1).

**Table 1:** Microinjection results of Nix construct.

Construct	No.embryos injected	Larvae Hatching %	Survival %	No. screened larvae	No. DsRed positive larva	No. transgenic lines	Transformation rate
pTRE-Nix/NixPro-tTA	2,316	509 (22%) *	509 (22%) ^†^	52,463	176	7	1.4%^‡^

*Percentage of injected embryos that hatched into L3- L4 larvae

†Percentage of injected embryos that hatched into adults

‡Percentage of independent transformed lines generated per fertile adult (assumes ~50% fertility)

### Transgenic Nix females are masculinized and do not blood feed

**Fig 2:**
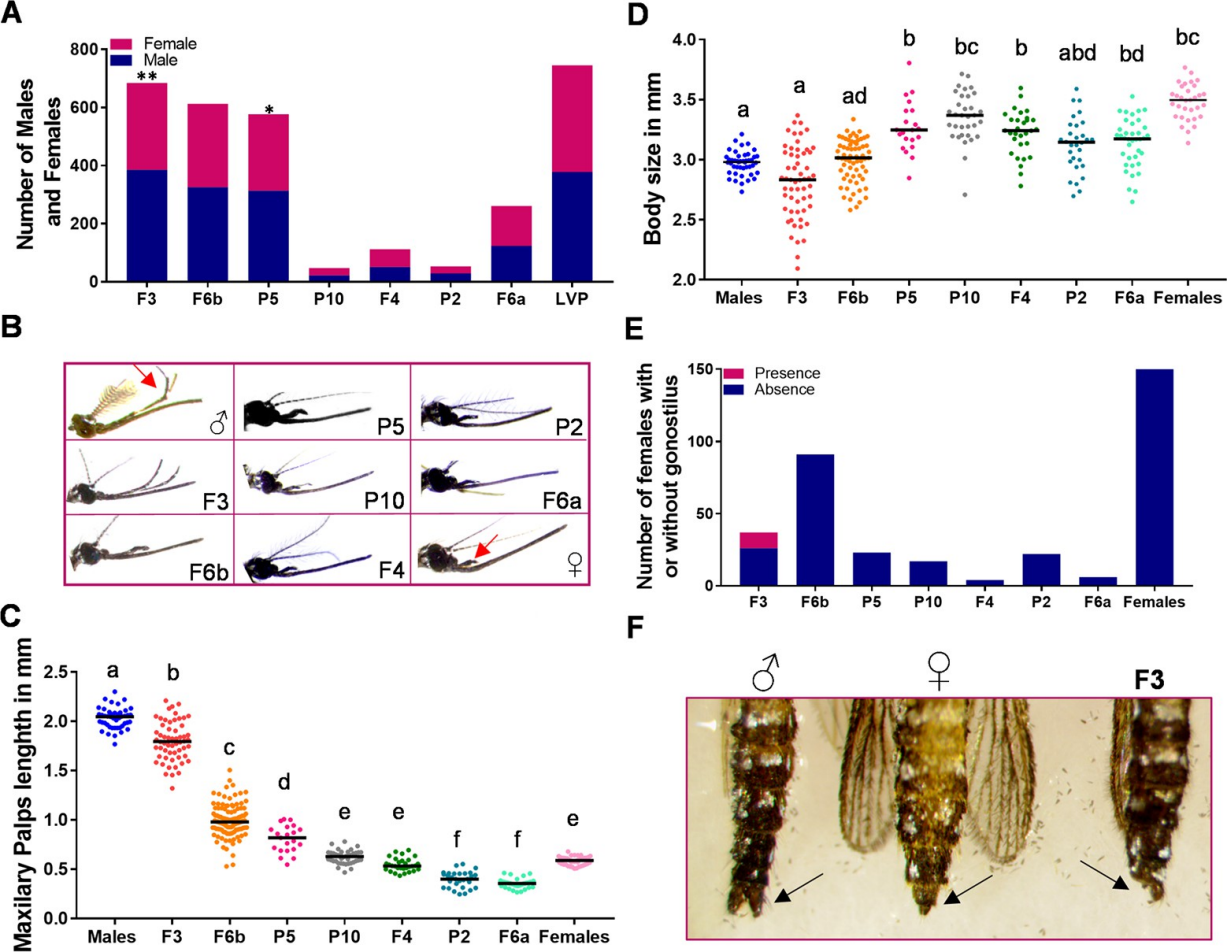
Intersex phenotypes in transgenic *Ae*. *aegypti* with Nix under the control of the TRE. (**A**) Sex ratio of each transgenic line. Binomial test was used to access statistical significance (**B**) Representative images of the head of the seven transgenic lines, male and female Liverpool strain depicting different maxillary palp lengths, red arrow indicating the maxillary palps from male and female wild type. (**C**) Maxillary palp size in millimeters from female mosquitoes of the seven transgenic lines, male and female from Liverpool in the absence of Dox. Bars represent the median. (**D**) Body size in millimeters from female mosquitoes of the seven transgenic lines, male and female from Liverpool strain in the absence of Dox. Bars represent the median. (**E**) Number of females from each transgenic line in absence of Dox that developed the male genitalia with the presence of one gonostilus (**F**) Pictures of F3 transgenic line, male and female Liverpool strain abdomen, the arrows are pointing to the genitalia depicting the presence (male), absence (female) or the presence of only one gonostilus (F3 line). (**C**) and (**D**) Numbers followed by different letters are statistically different P≤ 0.05 (Tukey’s test).

We hypothesized that transgenic mosquitoes expressing *Nix* early in embryo development would bias sex determination towards males and thus sought to evaluate the sex ratio from the transgenic males backcrossed to the LVP females. Only two of the seven transgenic lines, F3 and P5, presented a slight male bias (Fig 2A).

While most transgenic crosses with wildtype mosquitoes resulted in normal sex ratios, we observed that the female progeny from some transgenic line backcrosses displayed longer maxillary palps than LVP control females (Fig 2B). We hypothesized that although not fully converted those females were masculinized, as males have longer maxillary palps (Fig 2C). Maxillary palp size was subsequently used as a proxy for the degree of masculinization. We measured the maxillary palps from transgenic females from the seven transgenic lines obtained and three of them, F3, F6b and P5 had significantly longer maxillary palps in comparison to LVP females (Fig 2C). To determine the relative penetrance of the conversion phenotype we calculated Z scores for all samples based on the distribution of palp length in wild-type females. From this, we found that 85% of P5, 95% of F6b and 100% of F3 females displayed the long maxillary palp phenotype. The other four lines were found to either have the same maxillary palp size of the LVP females or smaller (Fig 2C). We also evaluated the body length of transgenic females, with lines F3 and F6b found to have body sizes that did not differ from control males (Fig 2D).

Our previous work expressing Nix either ectopically or under the control of its endogenous promoter had resulted in the partial or complete masculinization of the genitalia [10]. Thus, we investigated as to whether the genitalia from transgenic females remained normal or was masculinized in each transgenic strain. In 30% of the females from the F3 transgenic line we observed partial presence of one gonocoxite/gonostyli (Fig 2E). Fig 2F shows the partial presence of one gonocoxite/gonostyli in a masculinized female from the F3 line. The presence of gonocoxite/gonostyli structures was not observed in any other transgenic females, indicating that transgenic masculinization was limited to only a subset of individuals (Fig 2E).

**Fig 3:**
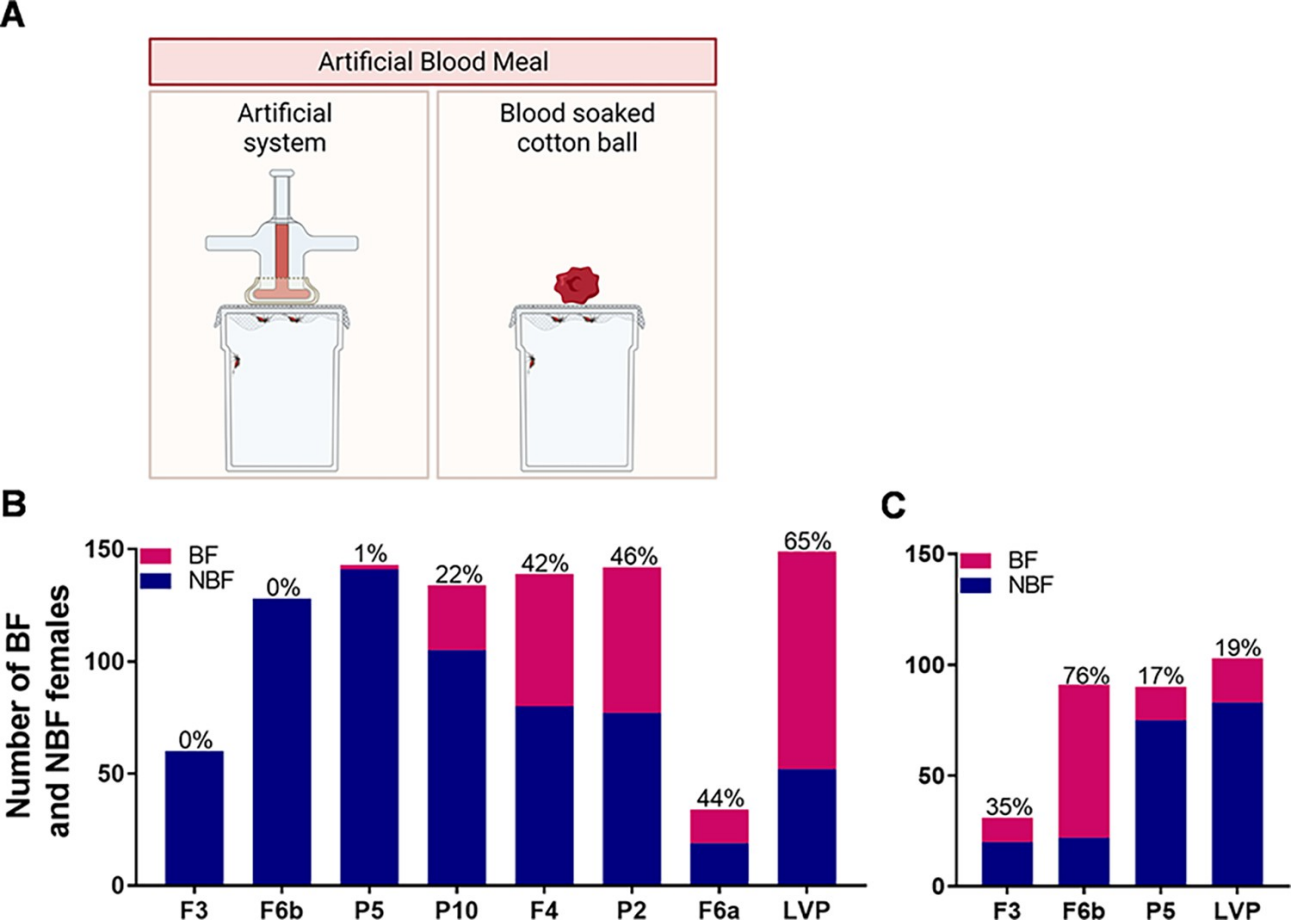
Transgenic females displaying higher levels of masculinization are not able to blood feed in the presence of a physical barrier. (**A**) Schematic representation of the two artificial blood meals offered, one with a physical barrier (artificial system) and one without the physical barrier (blood-soaked cotton ball) (**B**) Number of blood fed (BF) and non-blood fed (NBF) females from transgenic lines and Liverpool strain in the absence of Dox in the artificial blood meal system and (**C**) blood-soaked cotton ball. Percentages represent blood fed females. Illustrations were created using Biorender.com through a license to Texas A&M University.

In addition to changes in physical appearance and structure, we sought to determine if partially masculinized transgenic females were interested and capable of obtaining a bloodmeal (Fig 3A). Transgenic and LVP females were offered an artificial blood meal for 30 min and then scored for the presence of blood in the abdomen. None of the transgenic females from F3 and F6b lines and just 1% of the P5 transgenic females were able to blood feed in the artificial system (Fig 3B). The other four transgenic lines the blood feeding success ranged between 22% to 46%, while the LVP control was 65% (Fig 3B). While performing the blood feeding experiment, we noticed that the transgenic females from F3 and F6b, although not able to acquire a blood meal, did land on the artificial feeder and were persistently seeking the blood and trying to pierce through the parafilm. However, their proboscis bent and failed to penetrate the parafilm. We then offered a cotton ball soaked in warm blood (Fig 3A) to understand if the transgenic females were capable of ingesting blood in the absence of a physical barrier. After 30 min, 35% of the F3, 76% of the F6b, and 17% of the P5 transgenic females had obtained a blood meal, while only 19% from the non-transgenic counterparts were engorged (Fig 3C).

**Fig 4:**
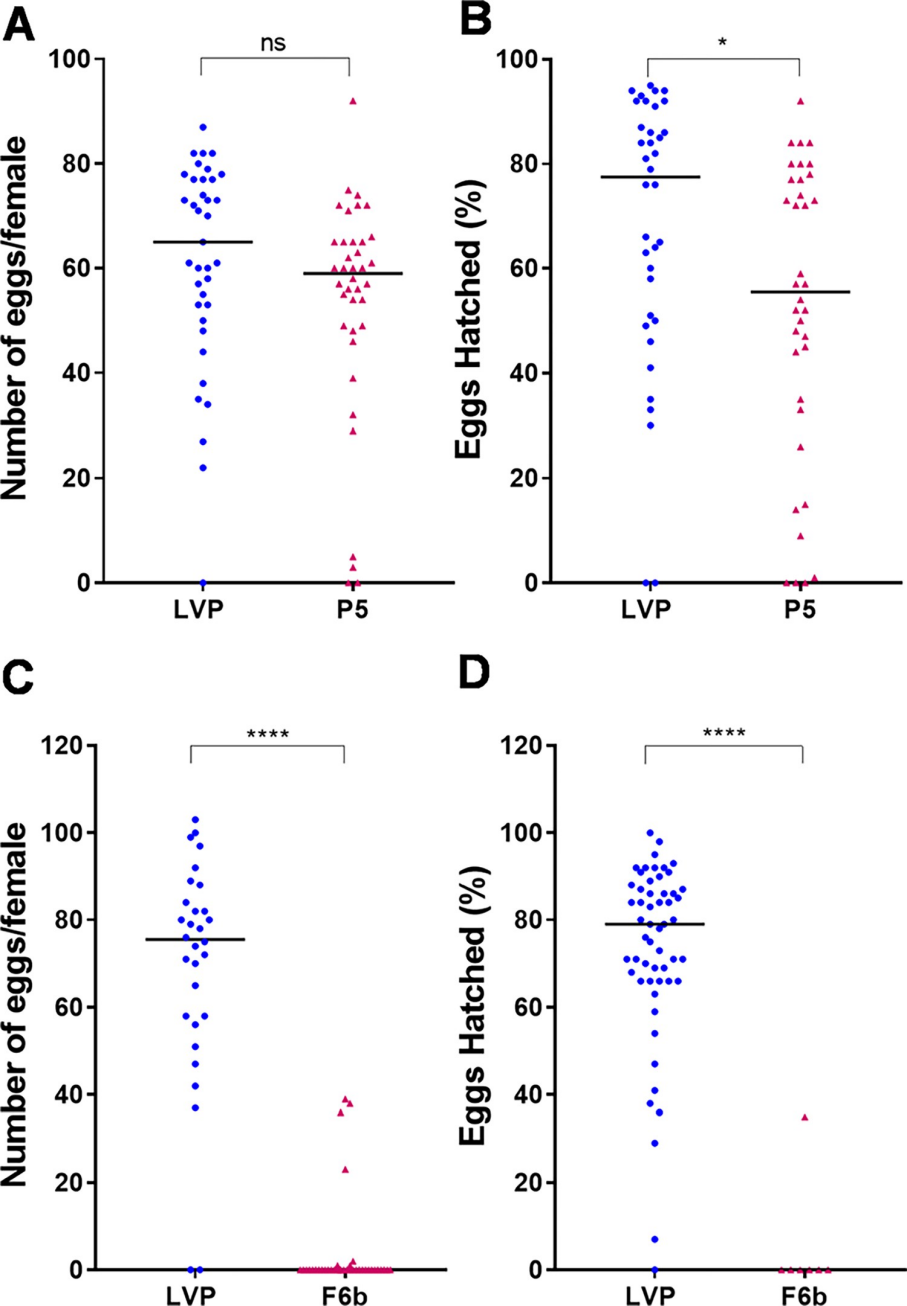
Reduced fecundity and fertility in masculinized transgenic females. Transgenic females were offered a blood meal in the artificial apparatus or through a warm blood-soaked cotton. Blood fed females were put into an Eagal plate and number of eggs per female (fecundity) and number of larvae per eggs (fertility) was scored. (**A**) Fecundity from P5, (**B**) fertility from P5 (**C**) fecundity from F6b, and (**B**) fertility from F6b transgenic lines. A Mann–Whitney U test was used to evaluate statistical significance. ns: P>0.05; ***: P<0.0001.

Since a subset of the partially masculinized F6b and P5 females were able to obtain a bloodmeal from the soaked cotton, we sought to determine whether they were competent to digest the blood and produce viable eggs. P5 transgenic females had no significant difference in fecundity in comparison to the non-transgenic counterparts (Fig 4A), however, a significant decrease in fertility was observed (Fig 4B). In contrast, in the more severely masculinized F6b line, only 6 out of 39 females were able to lay eggs, a significant difference in egg number in comparison to non-transgenic females (Fig 4C). From the 6 females that laid eggs only the eggs from one of them hatched but with significantly less larvae in comparison to non-transgenic females (Fig 4D). Finally, in the most heavily masculinized F3 transgenic line, blood fed females (29 total blood fed females in different experiments) were never able to lay any eggs.

### Ectopic Nix expression causes higher mortality in masculinized transgenic females

**Fig 5:**
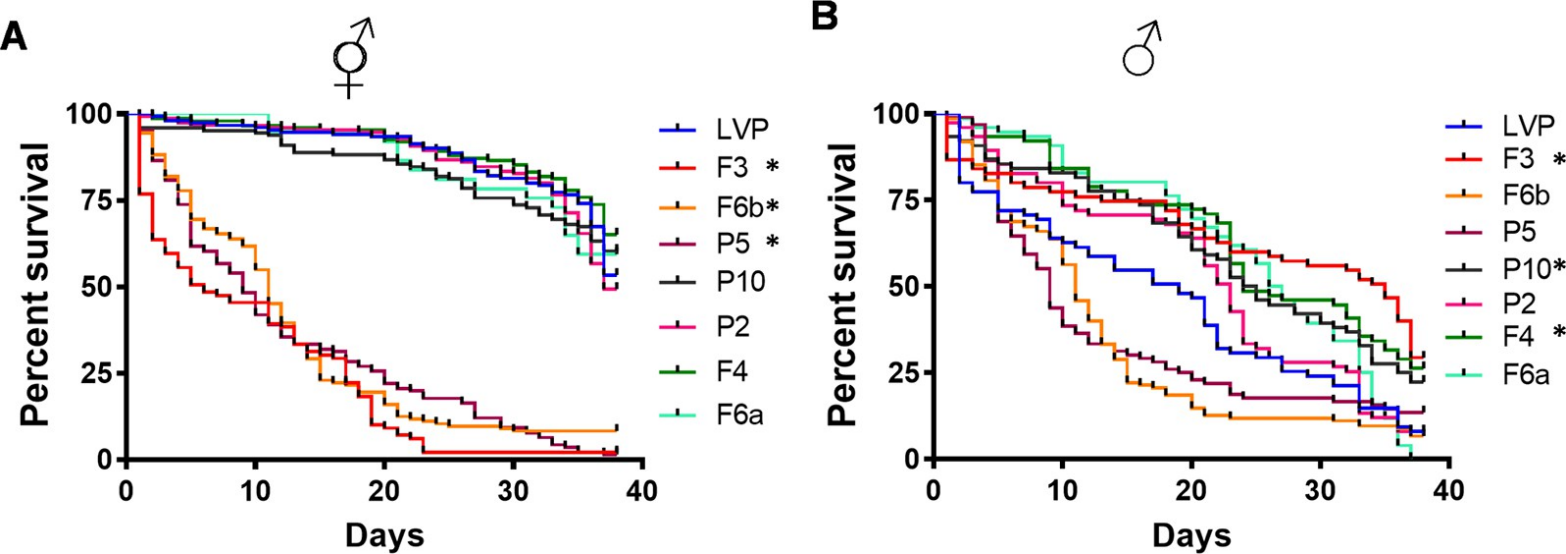
Transgenic females displaying higher masculinization have higher mortality. Survival curves of transgenic (**A**) transgenic females (at least 100 individuals per transgenic line) and (**B**) males (at least 75 individuals per transgenic line) and Liverpool strain (150 females and 75 males) in a total of 38 days of observation. Log-rank test was used to determine P values and statistical significance was determined after the Bonferroni method to correct for multiple testing. *: *P* < .07.

To determine the effect of partial masculinization on adult survival, we monitored the number of deaths each day for 38 days after adult emergence. Significantly higher mortality rates were observed in females from the transgenic lines F3, F6b, and P5 in comparison to the other transgenic lines and LVP females (Fig 5A). We then sought to evaluate if the extra copy of *Nix* would cause any effect on transgenic males. Unexpectedly, in three transgenic lines (F3, P10 and F4) male survival was significantly increased in comparison to the LVP control (Fig 5B). Though lines P5 and F6b appeared to die at a higher rate that the LVP control, this was not statistically significant.

### Doxycycline treatment partially restores normal phenotype in masculinized transgenic females

**Fig 6:**
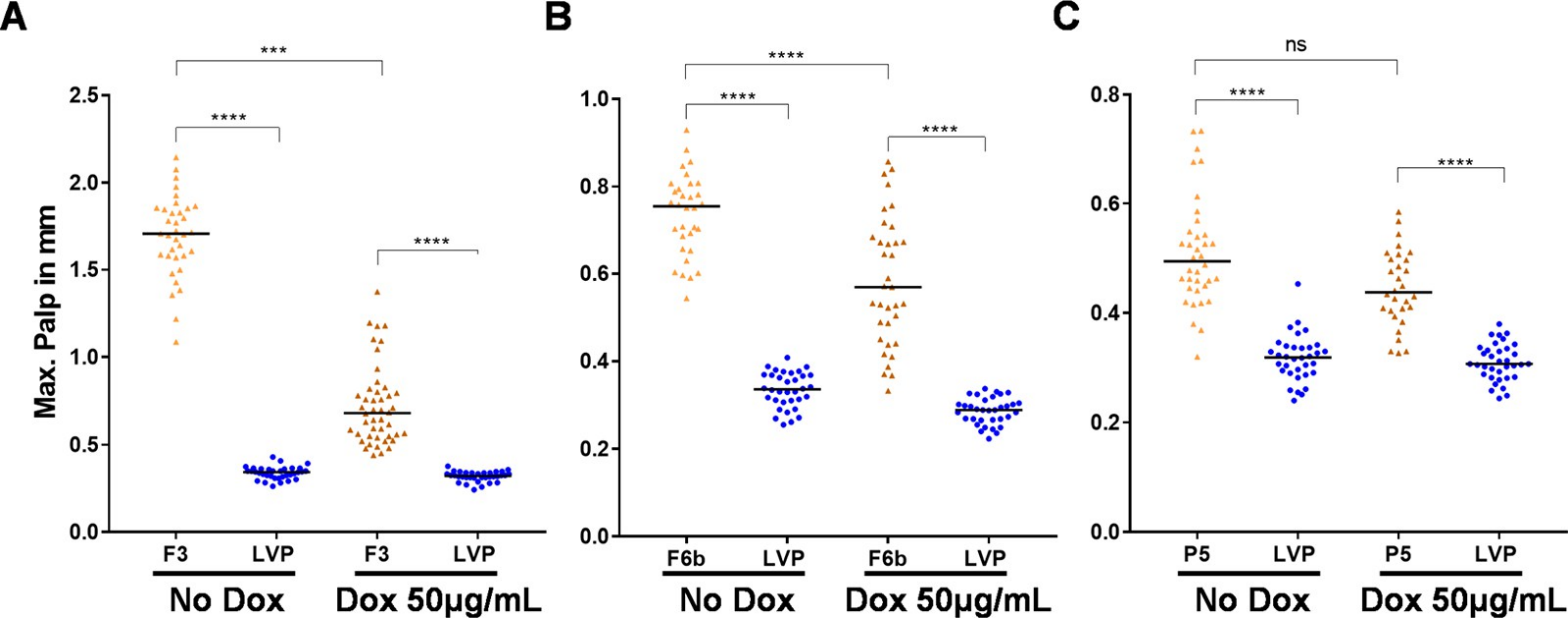
Doxycycline treatment partially restores maxillary palps size in transgenic females. Mosquitoes were reared in the absence or presence of 50ug/mL doxycycline and supplemented with SURE cells during larval and pupal stages and after emergence, adult pictures were taken and maxillary palps were measured from F3 (**A**), F6b (**B**) and P5 (**C**) transgenic lines and Liverpool strain. The bars from the maxillary palps measurements represent the median. A Mann–Whitney U test was used to evaluate statistical significance. ns: P>0.05; ***: P<0.0001.

We next questioned if the masculinization phenotype in the transgenic females from the F3, F6b and P5 lines could be reverted with treatment with doxycycline (Dox). Doxycycline was chosen as it is a known analog of tetracycline, however, a concentration ~10x lower was shown to be capable of producing the same blocking effect of tetracycline in the Tet-off system [27]. Initially, a concentration of 3μg/mL was chosen to rear the larvae and we proceeded to measure the maxillary palps from transgenic females treated or not treated with doxycycline. The maxillary palps from Dox-treated transgenic females from the F3 line were significantly shorter than the non-treated ones (S1 Fig), however, they were still significantly longer than LVP females (S1 Fig) and no difference in maxillary palps was observed between Dox-treated and non-treated females from the F6b line (S1 Fig). Since we could not completely restore the female phenotype of the transgenic females, we proceeded to increase the concentration of doxycycline to 50μg/mL. We noticed detrimental effects on larval development due to the high concentration of Dox, and to mitigate this effect we provided tet-resistant bacterial (SURE) cells to the larval water. Our intent was to recolonize the midgut microbiome with bacteria resistant to the antibiotic treatment and reestablish normal development for a more synchronous experiment (meaning that larvae on Dox and non Dox treatment would pupate almost at the same time). We observed a decrease of 4.5X, 1.2X, and 1.2X in maxillary palps of Dox treated females in comparison to non-treated ones for F3, F6b and P5 lines respectively (Fig 6A–6C). In fact, increasing the Dox concentration from 3μg/mL to 50μg/mL, decreased the maxillary palps of transgenic females in 1.7X and 1.2X in F3 and F6b lines (S2 Fig).

We also sought to evaluate if 50μg/mL Dox treatment could restore the ability of the transgenic females to blood feed. No difference in blood feeding success with the artificial feeder was observed between non-treated and treated transgenic females from F3, F6b and P5 (S3A–S3C Fig). We then evaluated if fecundity or fertility from the transgenic females from F3, F6b and P5 lines (that blood fed on artificial feeder and blood-soaked cotton) could be reestablished with the 50μg/mL Dox treatment, however no difference could be observed in Dox treated or no treated females (S4 Fig).

### Transgenic Nix mRNA expression and masculinization levels

**Fig 7:**
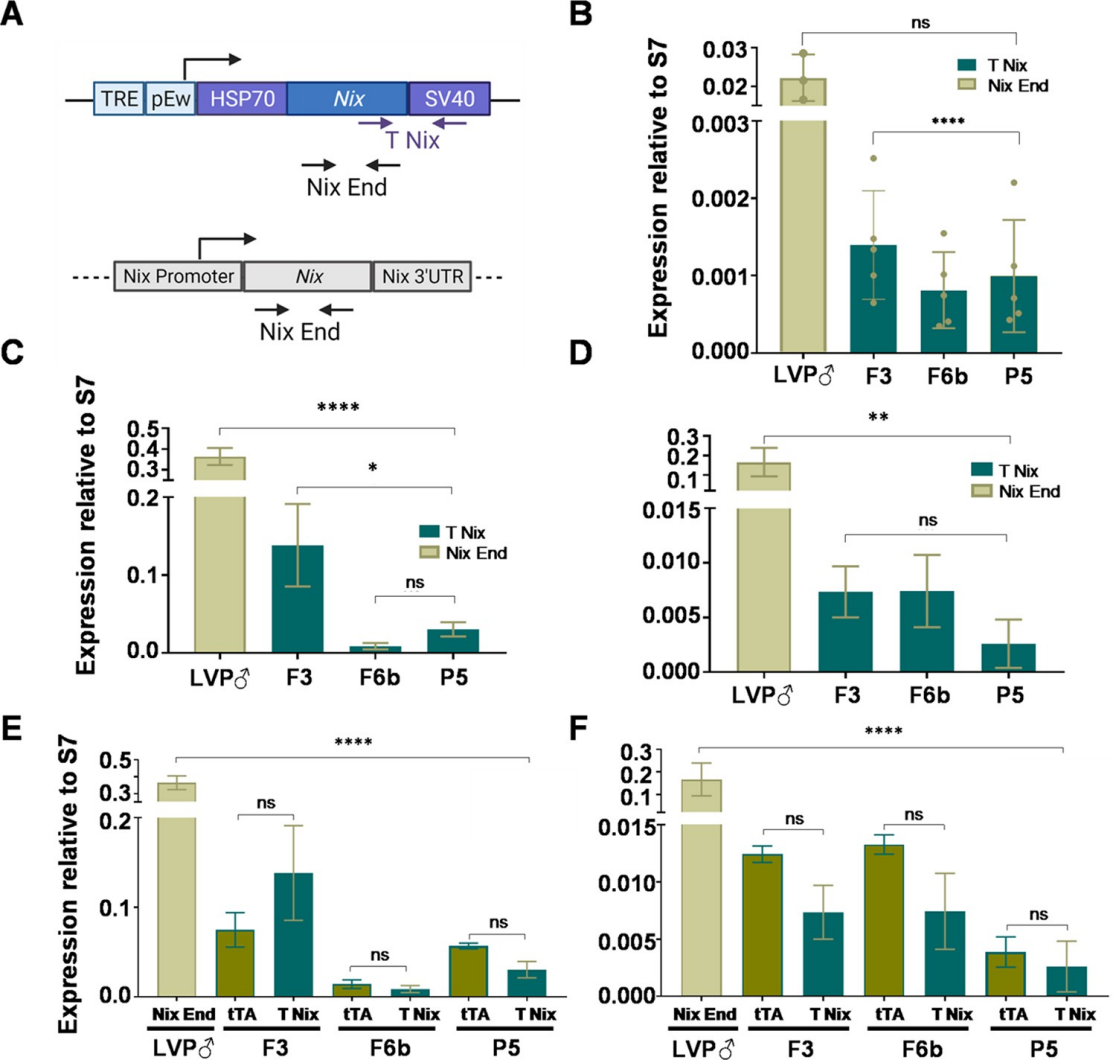
Transgenic *Nix* expression profile in transgenic mosquitoes. (**A**) Schematic representation of the pTRE-Nix/NixPro-tTA transgene (top) and endogenous *Nix* gene (bottom) and Nix T (specific for the transgene) and Nix End (binds on the *Nix* open reading frame on both the transgene and endogenous Nix) primer binding localization. (**B**) Quantification of endogenous or transgenic *Nix* transcripts in F3, F6b and P5 transgenic adult female mosquitoes and Liverpool males of at least 3 experiments containing 5 mosquitoes each. Quantification of accumulated transcript in the maxillary palps (**C, E**) and antennae (**D, F**) from 25–30 male Liverpool or female transgenic of transgenic *Nix* and *Nix* endogenous (**C-D**) and tTA and transgenic Nix (**E-F**). Columns represent the mean values and bars the standard. One-way ANOVA test was used to determine the statistical significance; ns: P>0.05; ****: P<0.0001. T Nix, transgenic *Nix* transcript; Nix End, endogenous *Nix* transcript, tTA, tetracycline transactivator.

Transgenic *Nix* expression was evaluated by RT-qPCR to verify if the different levels of masculinization observed among transgenic lines was due to differences in the transgene expression. Using two sets of primers (Fig 7A), transcript abundance in adult females was investigated, with no significant difference observed in transgenic *Nix* expression between F3, F6b and P5 transgenic lines, despite strong differences in masculinization (Fig 7B). However, they displayed significant lower levels in comparison to *Nix* expression in the wild type males (~15, ~44, and ~32 times lower in F3, F6b, and P5 respectively) (Fig 7B). We further assayed the expression levels of transgenic *Nix* and tTA in maxillary palps and antennae in the transgenic females and endogenous *Nix* expression in LVP males to determine if there were differences in somatic expression of transgenic *Nix* among the transgenic lines and LVP males as well as to investigate possible differences in tTA expression in relation to transgenic *Nix* that could indicate potential problems in the Tet-off system. LVP males expressed significantly more endogenous *Nix* than masculinized females expressed transgenic *Nix* in both tissues (Fig 7C and 7D). In the antennae, F3 females had higher expression of transgenic *Nix* in comparison to F6b and P5 (Fig 7C) despite the fact that we never observed masculine traits in this tissue in any transgenic lines. In the maxillary palps, where strong phenotypic differences were observed, no difference in transgenic *Nix* levels was detected among the lines (Fig 7D). Similar levels of expression of tTA and transgenic *Nix* were observed in both antennae and maxillary palps in the three transgenic lines, however tTA expression was substantially lower than endogenous *Nix* in LVP males (Fig 7E and 7F), indicating that the *Nix* promoter in the transgene failed to mirror the endogenous levels of expression but tTA properly activated transgenic *Nix* expression.

**Fig 8:**
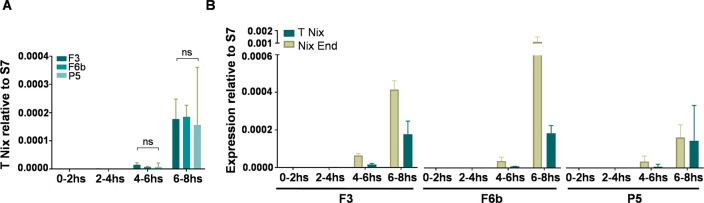
Transgenic and endogenous *Nix* expression profile in transgenic mosquitoes. Comparison of accumulated transgenic *Nix* transcript **(A)** and quantification of accumulated transgenic *Nix* transcript and *Nix* endogenous **(B)** from embryos collected 0-2h, 2-4h, 4-6h, 6-8hs after being laid from F3, F6b, and P5 transgenic lines. Columns represent the mean values and bars show the standard deviation of at least two independent experiments containing at least 50 embryos each. One-way ANOVA test was used to determine the statistical significance (**B**); ns: P>0.05; ***: P<0.0001.

The start of *Nix* transcription correlates to the beginning of the syncytial blastoderm stage right before sex determination about 4 hours after oviposition (Hall et al 2015). We evaluated the levels of transgenic *Nix* expression at four time points during embryonic development, spanning 0 to 8 hours after the embryos were laid from F3, F6b and P5 transgenic lines. Again, no difference in transgenic *Nix* expression was observed in any of the time points analyzed (Fig 8A). The expression of transgenic *Nix* replicated the pattern of expression of endogenous *Nix*, starting approximately at 4 hours and peaking at 6-8hs after oviposition in F3, F6b and P5 transgenic lines (Fig 8B), but the transgene failed to achieve the same intensity in expression as the endogenous gene expressing ~ 3 and ~1.3 times less at 4–6 hrs and 6–8 hrs respectively in the F3 line; ~4 and ~5 times less for F6b (Fig 8B). However, for line P5 although at the 4–6 hrs time point *Nix* was ~1.3 less expressed, no difference in expression was found in the 6–8 hrs time point (Fig 8B).

**Fig 9:**
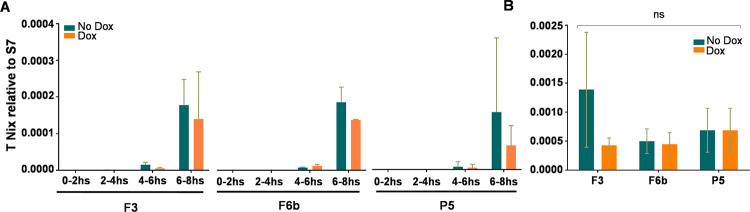
Impact of doxycycline on transgenic *Nix* expression. Quantification of accumulated transgenic *Nix* transcript treated and non-treated with 50μg/ml of Doxycycline from embryos collected 0-2h, 2-4h, 4-6h, 6-8hs after being laid from F3, F6b, and P5. (**A**) Transgenic lines and (**B**) Adult female mosquitoes. Columns represent the mean values and bars the standard deviation (**A-B**) of at least two independent experiments containing at least 50 embryos each (**A**) and 3 independent experiments (**B**). Multiple t test was used to determine the statistical significance (**B**); ns: P>0.05; ***: P<0.0001.

To investigate the impact of doxycycline treatment on transgenic *Nix* expression in embryonic stages, LVP females mated with transgenic males from F3, F6b and P5 lines were kept with sucrose with or without 50μg/ml of doxycycline until oviposition and eggs were collected in containers with water with or without 50μg/ml of doxycycline. Minimal changes in expression levels of transgenic *Nix* were observed in embryos treated and non-treated with doxycycline (Fig 9A). For the analyses in adult mosquitoes, the same treatment as the embryos was performed, but larval stages were supplemented with SURE cells with or without 50μg/ml of doxycycline until pupal stages. No difference in expression levels of transgenic *Nix* was observed in adult mosquitoes treated and non-treated with doxycycline (Fig 9B). Overall, doxycycline treatment did not appear to impact global expression of the *Nix* transgene.

### Transgenic Nix insertion sites are unlikely to contribute directly to the varied sex conversion phenotypes

Splinkerette PCR was performed to confirm the integration events from the F3, F6b and P5 transgenic lines. Though unlikely since each event represents an independent random integration, we also sought to rule out integration site as a potential cause of intersex phenotypes (by integrating near or within known genes involved in the development of sexually dimorphic structures). The sequences flanking the transposable element were determined (S5 Fig). For all insertions, the expected tetranucleotide TTAA was identified between piggyBac left arm and the identified sequence, a fingerprint from piggyBac transposase-mediated insertion [28] (S1 Fig). Flanking sequences from the transgene in line F3 matched the 5’UTR one of 4 isoforms of a gene AAEL005016. This gene contains conserved domains of the malectin protein, which is a membrane-anchored endoplasmic reticulum protein highly conserved in animals and is implicated in early steps of protein N-glycosylation [29].The insertion site for the transposon in line F6b mapped to the middle of a 10kb intron present in the gene AAEL000128. This is predicted to be a cyclin-like protein similar to Retinoblastoma-associated protein, and is thus expected to be involved in the regulation of the cell cycle. Finally, the best match to the P5 flanking region was in the 157kb annotated intron of the lim homeobox gene, AAEL007120. None of the insertions appear to disrupt the coding regions of the respective nearby genes, and none of the genes have an association with sexually dimorphic development, suggesting that the phenotypes we observed are better explained by our ectopic expression of Nix, and not by a fortuitous landing site.

## Discussion

The necessity for *Ae*. *aegypti* female mosquitoes to acquire a blood meal for egg production serves as a bridge from pathogens like dengue, Zika, and chikungunya viruses to humans, and this fact defines its ability to transmit these disease-causing agents. Because of this behavior, genetic population suppression approaches have focused on eliminating females or shifting sex ratios towards harmless males [9,10,30–32]. This could be achieved in *Ae*. *aegypti* by manipulating the mosquito genome, introducing a transgene that conditionally expresses the male determining factor Nix. The Nix-based sex conversion strategy could also cut the costs associated with rearing females in male mass release programs [33]. In the present study, seven transgenic lines containing a transgene that conditionally expresses *Nix* under the Tet-off system were successfully obtained where three lines (F3, F6b and P5) displayed different levels of masculinization, determined by male-specific morphological features. First, longer maxillary palps were observed in all three lines. Body size was also diminished in F3 and F6b transgenic females displaying the same size as the wild type males. About 30% of F3 females had a partial gonocoxite/gonostyli, appendages of the male genitalia. We found that from all parameters analyzed the maxillary palp size was the most reliable and thus it was used as a proxy for determining different levels of masculinization. According to this criterion, F3 was the most masculinized followed by F6b and P5. In contrast, other strongly sexually dimorphic traits such as antennae structure generally were not substantially masculinized, highlighting the mosaic nature of the partial conversions through the Tet-off system. The other four transgenic lines obtained did not display any masculine traits, possibly due to position effects caused by the different genomic environment of the insertion sites and therefore were not further analyzed.

Similar masculinization phenotypes were also observed in *Ae*. *aegypti* females where ectopic *Nix* expression driven by polyubiquitin promoter was used [5] and in *An*. *gambiae* with the ectopic expression of *Yob* (male determining factor) driven by the vas2 promoter [6,34]. In contrast, complete female-to-male conversion was achieved using the endogenous promoter to express *Nix* in *Ae*.*aegypti* and *Ae*. *albopictus* [10,35], indicating that using a promoter region other than the endogenous one can be potentially detrimental for effective male sex determination.

Importantly, highly masculinized females could not blood feed whenever there was a physical barrier that required piercing by the proboscis, as was seen using an artificial blood feeding system. Once this barrier was removed (by offering a warm soaked cotton), the females were able to acquire blood, suggesting that they did not lose their blood-seeking behavior, although further studies would be required to confirm this. In agreement with that is our observation that those females were actively trying to blood feed on the artificial apparatus, however, their proboscis were not able to pierce the parafilm and they would bend whenever they tried to push their heads against it. Anomalies in proboscis structure were also observed in intersex females of *An*. *gambiae* where the female-specific exon of the *doublesex* gene was targeted by CRISPR/Cas9 [31]. Interestingly, two of the most masculinized lines had a higher blood feeding success in comparison to LVP females using the warmed soaked cotton, leading us to question if transgenic mosquitoes lack key mediators of heat seeking. Female *An*. *gambiae* mosquitoes with a knockout of Ir21a, an ionotropic receptor, had greatly reduced heat seeking capability mostly through lack of cooling-mediated repulsion [36]. Since host-seeking in mosquitoes is characterized by several modes of activities initiated with long-range chemosensory and visual clues combined with short-ranged thermosensory clues [37], it is possible that transgenic females could have lost the ability to repulse cooler temperatures and were still attracted to warmed soaked cotton through other cues while LVP females lost the interest in the blood due to lower temperatures. However, more evidence would be required to support this possible explanation. Not surprisingly, fecundity and fertility were impacted to different levels and correlated with the levels of masculinization. The F3 line was never able to produce eggs, while F6b had significantly lower numbers, but P5 produced the same number of eggs as the wild type females. Following the same trend, a high impact on fertility was observed in F6b and a mild impact was observed with P5 females.

Indeed we found a male bias in line F3 and P5 but not in F6b, however we never observed any males that were unable to fly, which would be expected in a complete conversion event, as those females would lack the *myo-sex* gene, a myosin heavy chain gene, required specifically for male flight and associated with the M-locus [10], and would presumably fail to express the female myosin gene, *myo-fem* [38]. The fact that we observed significantly higher mortality in the transgenic females from the most masculinized lines in comparison to the wild-type control but not in the other transgenic lines suggests that male bias found in F3 and P5 could be due to a lethality effect by ectopic expression of Nix or tTA. Autosomal expression of the M-factor *Guy1* in *An*. *stephensi* caused 100% lethality in females due to the involvement of *Guy1* in the regulation in dosage compensation, a mechanism that ensures similar levels of X-linked gene expression in heteromorphic sex chromosomes organisms [9,39]. This is unlikely for *Ae*. *aegypti* since it contains homomorphic sex-determining chromosomes [40–42]. While it is possible that in the F3 line the insertion of the transgene disrupted splicing or expression of a protein containing a domain that could be linked to protein N-glycosylation process, no gene disruption could be detected for P5 and F6b. In addition, only highly masculinized females displayed higher mortality and not the lines that failed to display masculinization.

Surprisingly, the levels of transgenic *Nix* expression in embryos, maxillary palps, and in adults did not correlate with different levels of masculinization, with no significant difference observed among transgenic lines. It is possible that such expression differences only manifest at other times during development, for example in the pupal stages where those structures are being formed. Antennae, on the other hand, did show a significant difference in transgenic *Nix* expression, being more expressed in the F3 line than F6b and P5 despite the fact that this structure did not display a male characteristic in any of the masculinized lines. It is possible that there could be a dose dependent threshold in *Nix* expression for the development of male characteristics, and while expression levels differed between transgenic lines none of them reached this essential threshold. In fact, we found that transgenic lines expressed significantly less transgenic *Nix* than the endogenous gene both in embryos and in adults (with the exception of P5) possibly explaining why the females did not fully convert into males as well.

While we have shown previously that ectopic expression of the *Nix* under its own promoter is sufficient to convert females into males [10], the addition here of the Tet-off system somehow diminished the levels of transgenic *Nix*, possibly below threshold levels, compromising full conversion. In the split system, transcription factors must activate the *Nix* promoter to express the tTA, followed by tTA activation of *Nix*. Thus, decreased expression may be due to a failure to produce sufficient tTA, failure of tTA to activate Nix expression, or both. The observed 17-fold lower level of expression of tTA as compared with endogenous *Nix* in the maxillary palps and 7-fold difference in the antennae, suggests that insufficient expression of tTA is likely the most important factor in the incomplete conversion phenotypes we observed. Reduced expression of tTA from the endogenous *Nix* promoter could have been due to position effects caused by the surrounding genomic environment, or to the absence of key enhancers present in the coding region of endogenous *Nix* gene that would be missing in the tTA construct. In Aryan et al (2020) [10], we obtained two transgenic strains, and both showed complete conversion. Here, we obtained seven strains, and none did. While this could just be bad luck, it could also be a form of selection bias in that only those lines with reduced expression of tTA could survive and be established. We observed similar biases in only obtaining low-expression tTA lines using the maternal/early zygotic-specific bZIP promoter [43]. Thus, achieving repressible control of *Nix* expression may require other, less toxic systems.

Doxycycline treatment only partially restored maxillary palp size even at the highest concentrations. It failed to recover blood feeding ability in transgenic lines and fecundity and fertility in line F6b, but was able to restore fertility in line P5. This partial recovery of the wild type phenotype is not surprising as other authors using the Tet-off system faced the same issue when attempting to rescue a flying phenotype from flightless transgenic mosquitoes [44]. We also observed that the different transgenic lines responded differently to the doxycycline treatment, possibly due to chromatin environment in the transgene insertion. The overall levels of Nix expression did not differ from Dox-treated and non-treated transgenic adult females and embryos at the different time points as revealed by RT-qPCR although we did observe the partial recovery in the maxillary palp size.

Despite the recovery of strong partial sex conversion phenotypes that resulted in the complete inability of several strains to blood feed, it is evident that the system presented here requires further refinement in regards to both gene activation (resulting in more complete conversion) and repression (allowing the development of homozygous strains that can be maintained in the factory setting). The use of different gene regulators could potentially improve and provide tighter regulation of the gene of interest. In addition, increasing the number of transgenic lines could offer genomic insertions where the position effect could favor gene regulation. Successful genetic population suppression strategies are of prime importance for controlling vector-borne diseases resulting in vector elimination and blocking disease transmission. The data presented here support the development and improvement of Nix-based sex conversion strategies.

## Supporting information

S1 Table qPCR Primers and efficiency. (XLSX)

S1 Fig Larval treatment with 3ug/mL of doxycycline does not restore maxillary palps size in transgenic females. Mosquitoes were reared in the absence or presence of 3ug/mL doxycycline during larval and pupal stages and after emergence, adult pictures were taken and maxillary palps were measured from F3 (**A**), F6b (**B**) transgenic lines, and Liverpool strain. The bars from the maxillary palps measurements represent the median. A Mann–Whitney U test was used to evaluate statistical significance. ns: P>0.05; ***: P<0.0001.
(TIF)

S2 Fig Different doxycycline concentration treatment comparison in the maxillary palps size in transgenic females. Mosquitoes were reared in the absence (No Dox), presence of 3ug/mL or 50ug/mL doxycycline during larval and pupal stages and after emergence, adult pictures were taken and maxillary palps were measured from F3 (**A**), F6b (**B**) transgenic lines and Liverpool strain male and females. The bars from the maxillary palps measurements represent the median. A Mann–Whitney U test was used to evaluate statistical significance. ns: P>0.05; ***: P<0.0001.
(TIF)

S3 Fig Blood feeding success in transgenic and Liverpool females in the presence or absence of doxycycline. Mosquitoes were reared in the absence (No Dox), or presence of 50ug/mL doxycycline during larval and pupal stages, supplemented with SURE cells and after emergence, females were offered a blood meal in the artificial apparatus or through a warm blood-soaked cotton. Females were scored as blood fed (BF) with the presence of blood in the abdomen and non-blood fed (NBF) with no blood visible in the abdomen. (**A**) Females from F3, (**B**) F6b and (**C**) P5 transgenic lines.
(TIF)

S4 Fig Fecundity and fertility in transgenic and Liverpool females in the presence or absence of doxycycline. Mosquitoes were reared in the absence (No Dox), or presence of 50ug/mL doxycycline (Dox) during larval and pupal stages, supplemented with SURE cells and after emergence, females were offered a blood meal in the artificial apparatus or through a warm blood-soaked cotton. Blood fed females were put into an Eagal plate and number of eggs per female (fecundity) and number of larvae per eggs (fertility) was scored. (**A**) Fecundity from P5, (**B**) fertility from P5 (**C**) fecundity from F6b, and (**B**) fertility from F6b transgenic lines. A Mann–Whitney U test was used to evaluate statistical significance. ns: P>0.05; ***: P<0.0001.
(TIF)

S5 Fig Splinkerette PCR. Splinkerette PCR was performed using genomic DNA extracted from F3 (**A**), F6b (**B**), and P5 (**C**) transgenic lines. The blue arrow indicates the sequence from the piggyBac left arm, in red the SPLNK primer sequence, in bold the tetra nucleotide TTAA that highlights the point of transgene insertion, and underline is the genomic sequence. Bottom panel from each figure is a schematic representation of the genes where the transgene landed from each line, the black arrow indicates the position in the gene where the transgene was inserted.
(TIF)
