# Supplementary figures and images for: Partial masculinization of Aedes aegypti females by conditional expression of Nix
Source: PLoS Negl Trop Dis. 2022 Jul 1;16(7):e0010598. doi: 10.1371/journal.pntd.0010598 (PMC9307153; doi:10.1371/journal.pntd.0010598)

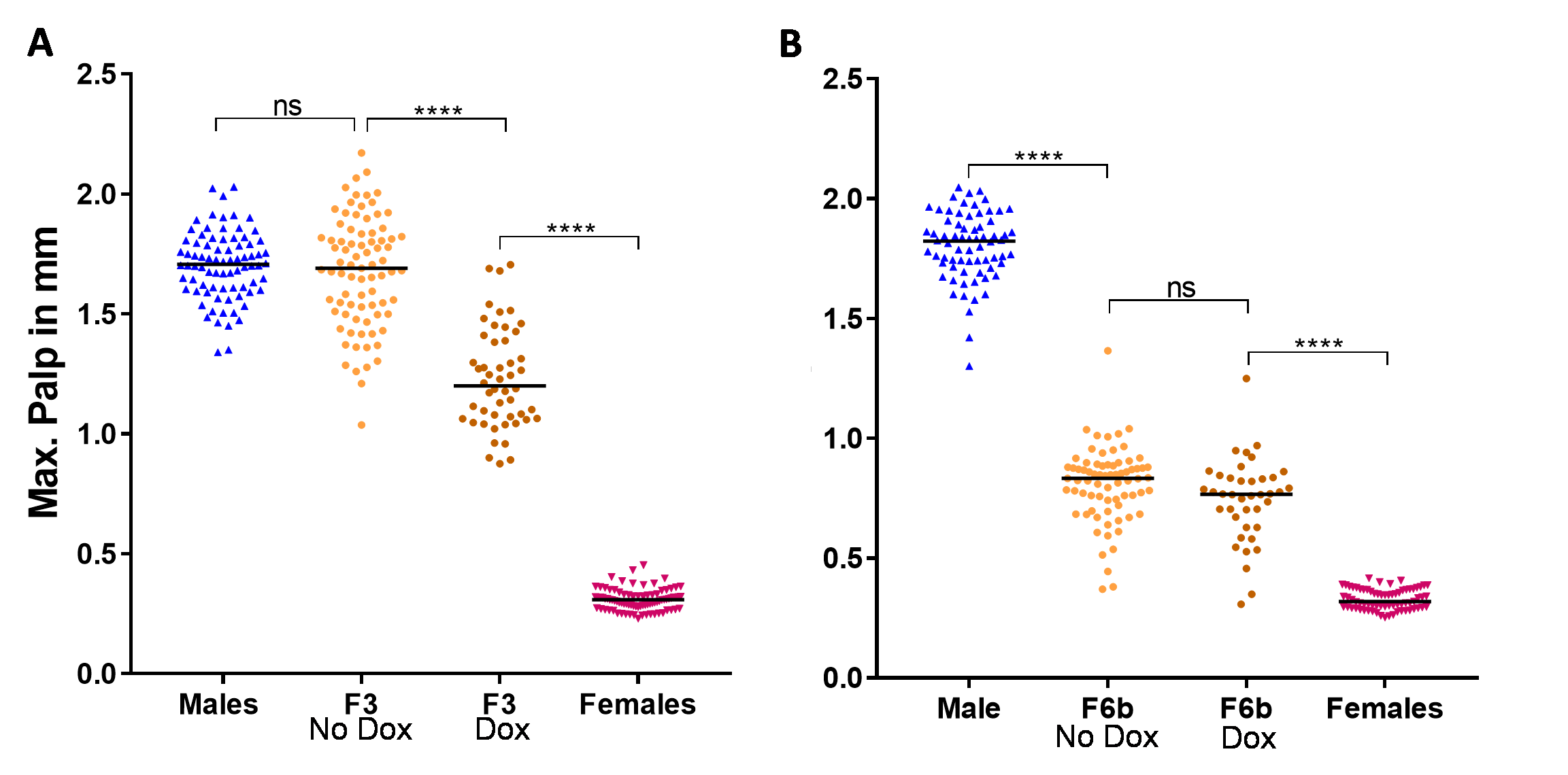

Supplement: S1 Fig — Mosquitoes were reared in the absence or presence of 3ug/mL doxycycline during larval and pupal stages and after emergence, adult pictures were taken and maxillary palps were measured from F3 (A), F6b (B) transgenic lines, and Liverpool strain. The bars from the maxillary palps measurements represent the median. A Mann–Whitney U test was used to evaluate statistical significance. ns: P>0.05; ***: P<0.0001. (TIF) [file pntd.0010598.s002.tif]

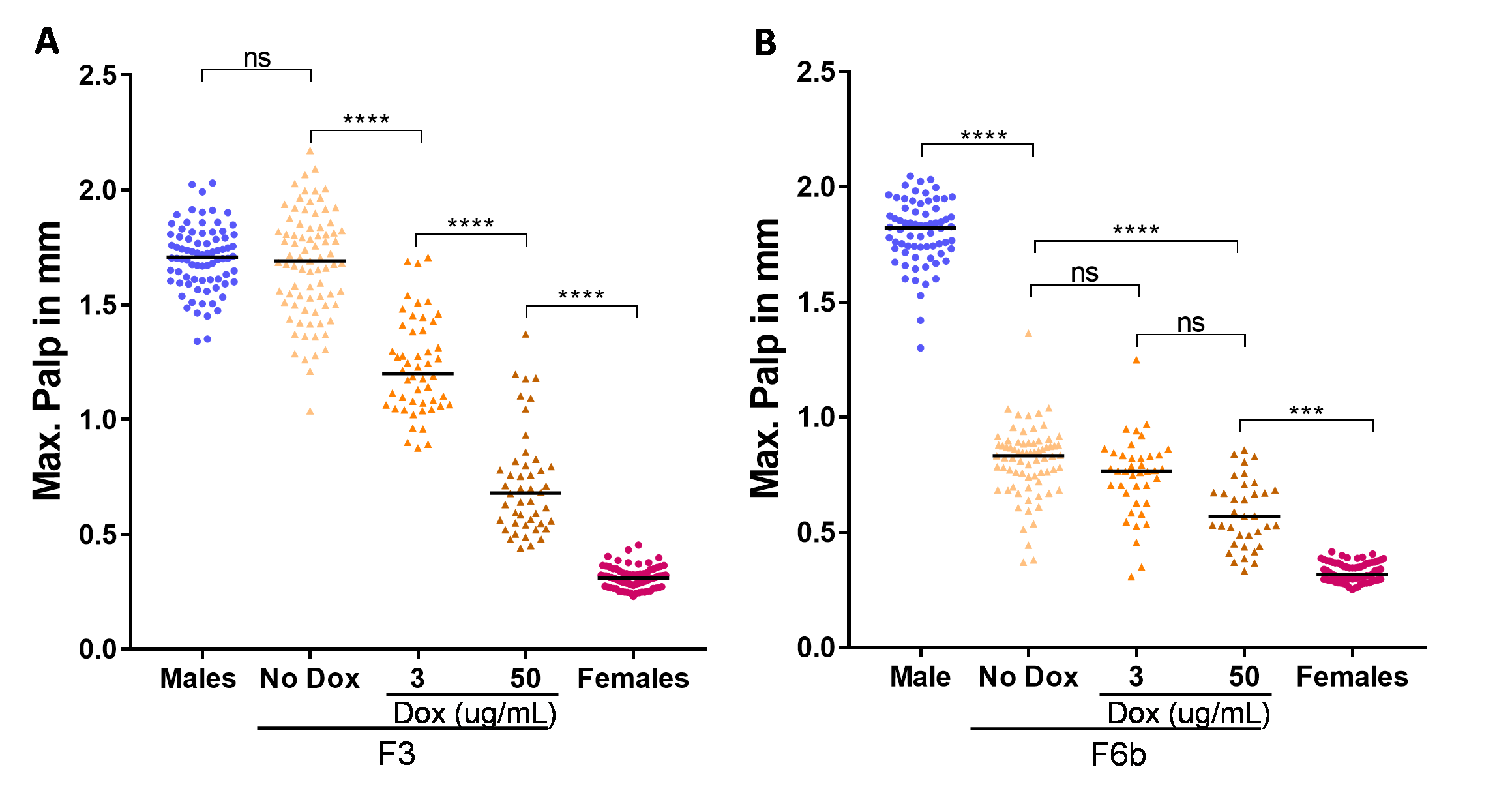

Supplement: S2 Fig — Mosquitoes were reared in the absence (No Dox), presence of 3ug/mL or 50ug/mL doxycycline during larval and pupal stages and after emergence, adult pictures were taken and maxillary palps were measured from F3 (A), F6b (B) transgenic lines and Liverpool strain male and females. The bars from the maxillary palps measurements represent the median. A Mann–Whitney U test was used to evaluate statistical significance. ns: P>0.05; ***: P<0.0001. (TIF) [file pntd.0010598.s003.tif]

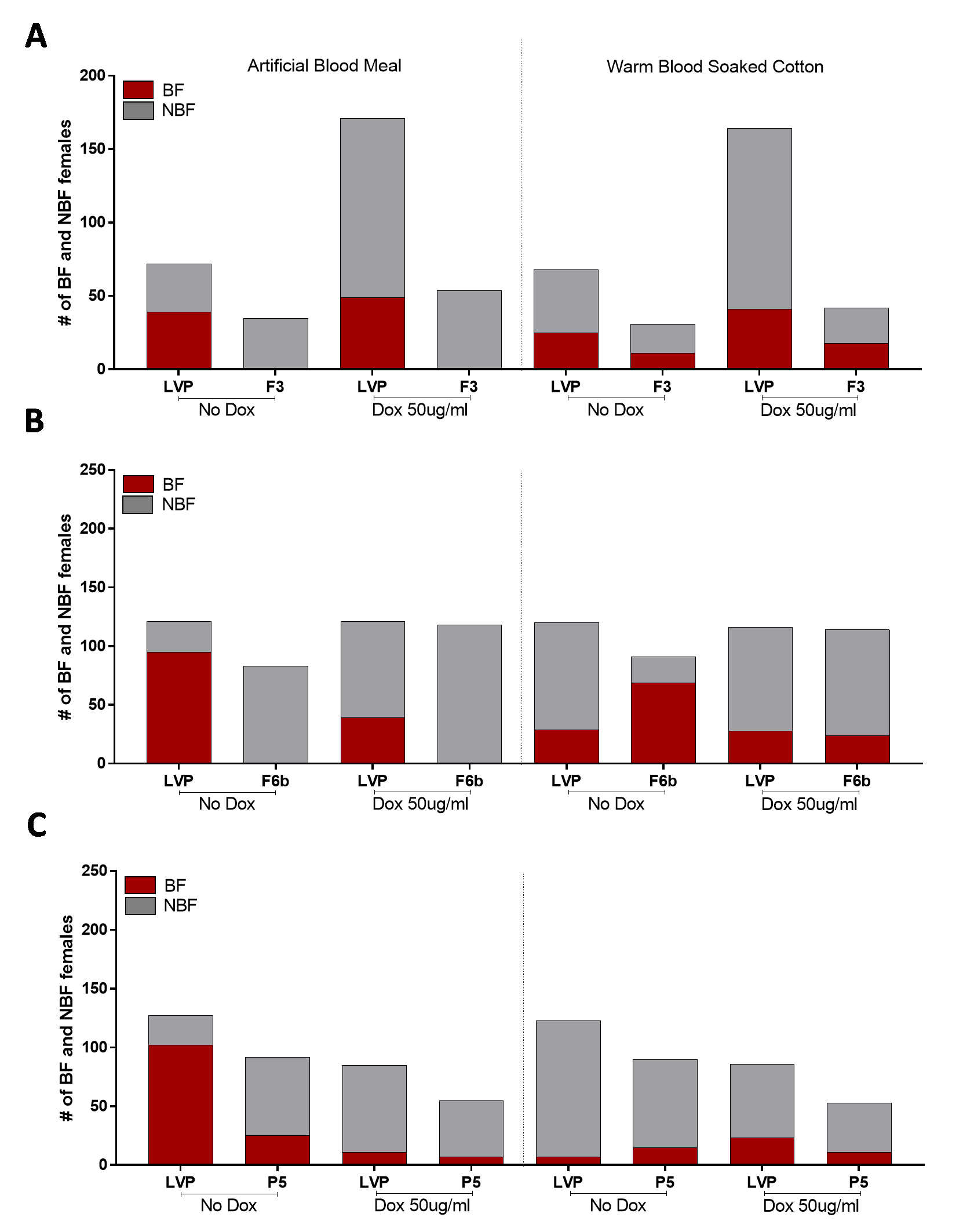

Supplement: S3 Fig — Mosquitoes were reared in the absence (No Dox), or presence of 50ug/mL doxycycline during larval and pupal stages, supplemented with SURE cells and after emergence, females were offered a blood meal in the artificial apparatus or through a warm blood-soaked cotton. Females were scored as blood fed (BF) with the presence of blood in the abdomen and non-blood fed (NBF) with no blood visible in the abdomen. (A) Females from F3, (B) F6b and (C) P5 transgenic lines. (TIF) [file pntd.0010598.s004.tif]

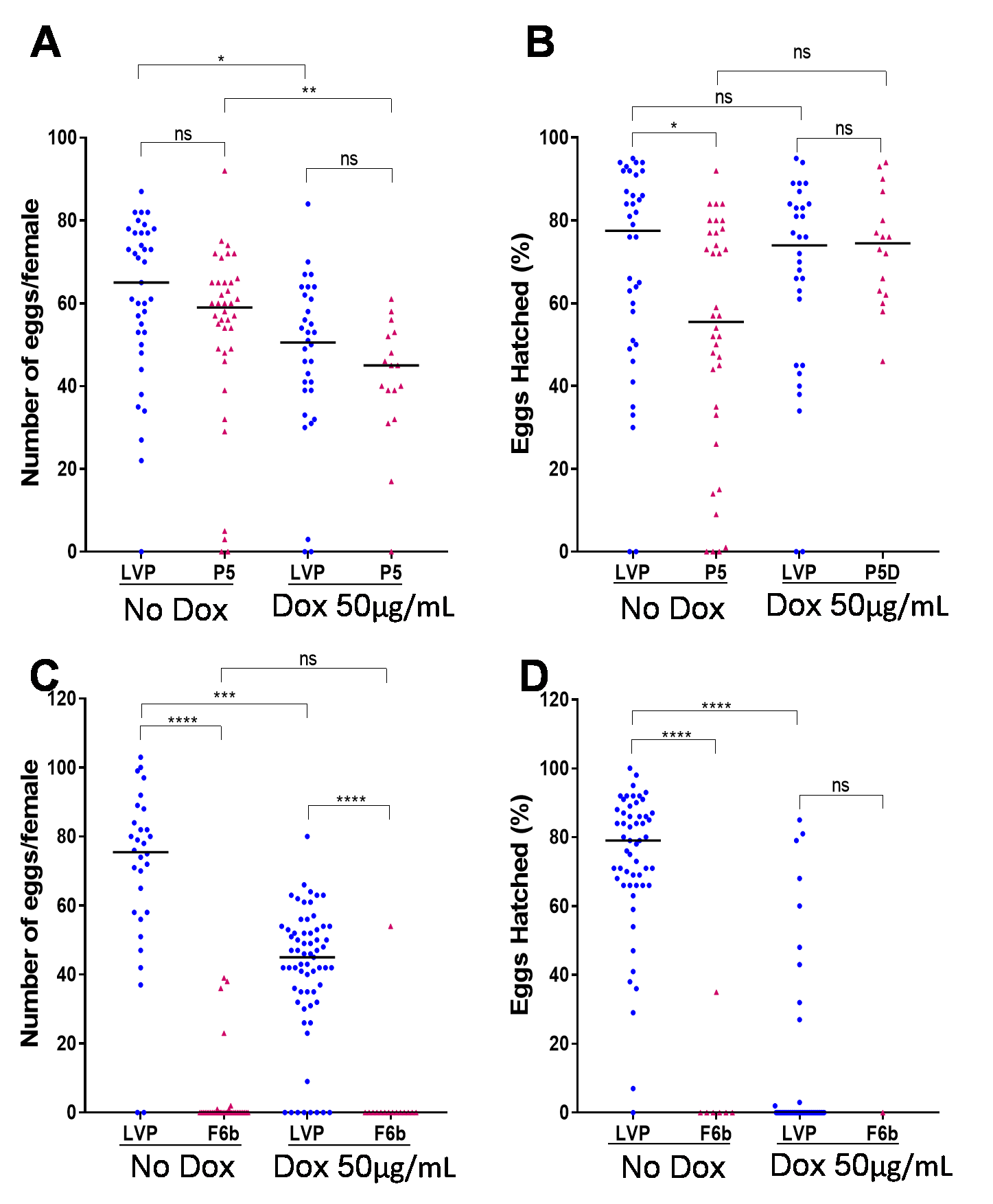

Supplement: S4 Fig — Mosquitoes were reared in the absence (No Dox), or presence of 50ug/mL doxycycline (Dox) during larval and pupal stages, supplemented with SURE cells and after emergence, females were offered a blood meal in the artificial apparatus or through a warm blood-soaked cotton. Blood fed females were put into an Eagal plate and number of eggs per female (fecundity) and number of larvae per eggs (fertility) was scored. (A) Fecundity from P5, (B) fertility from P5 (C) fecundity from F6b, and (B) fertility from F6b transgenic lines. A Mann–Whitney U test was used to evaluate statistical significance. ns: P>0.05; ***: P<0.0001. (TIF) [file pntd.0010598.s005.tif]

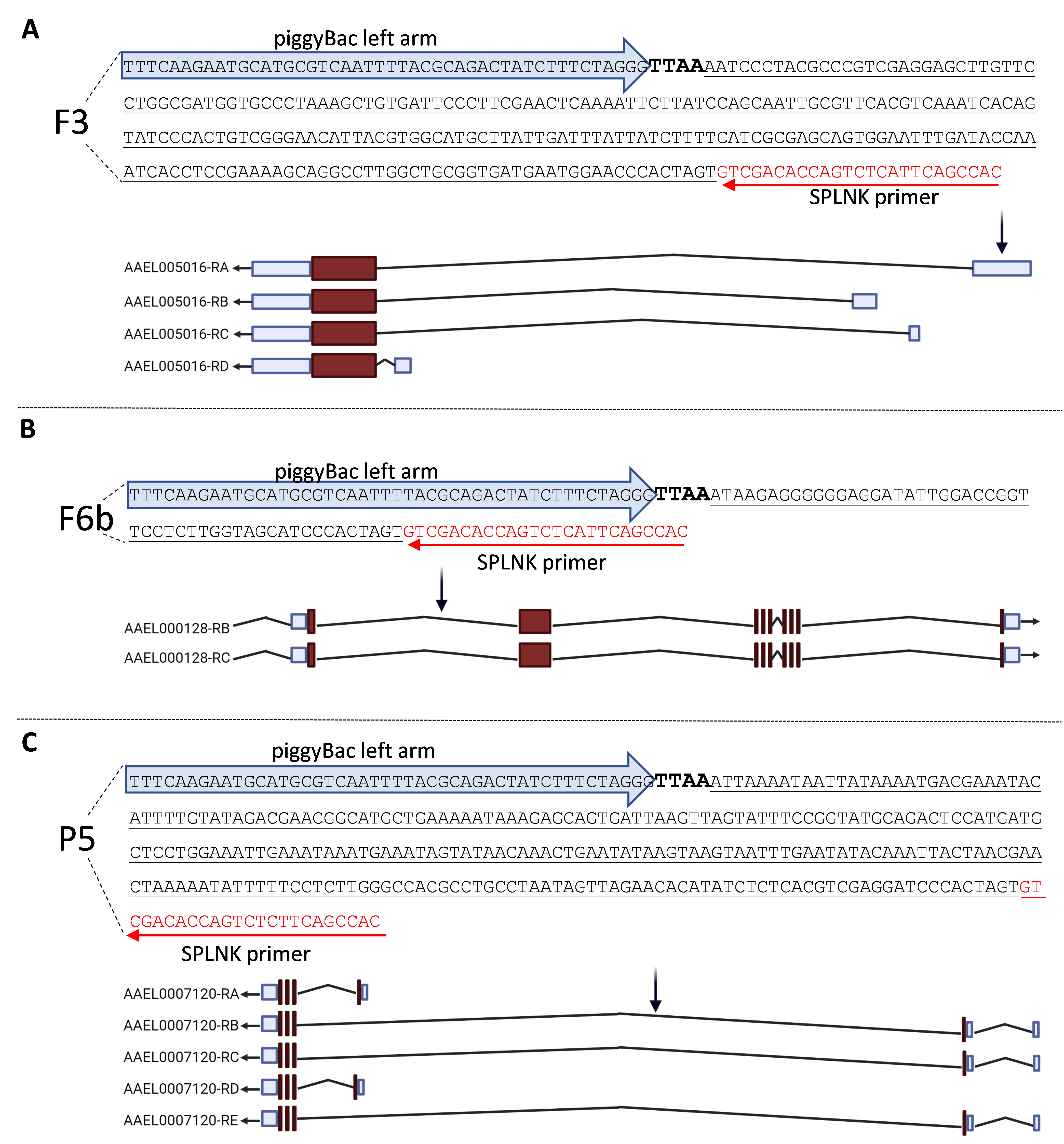

Supplement: S5 Fig — Splinkerette PCR was performed using genomic DNA extracted from F3 (A), F6b (B), and P5 (C) transgenic lines. The blue arrow indicates the sequence from the piggyBac left arm, in red the SPLNK primer sequence, in bold the tetra nucleotide TTAA that highlights the point of transgene insertion, and underline is the genomic sequence. Bottom panel from each figure is a schematic representation of the genes where the transgene landed from each line, the black arrow indicates the position in the gene where the transgene was inserted. (TIF) [file pntd.0010598.s006.tif]
